# Prevalence of preterm birth and associated factors among mothers who gave birth in public hospitals of east Gojjam zone, Ethiopia

**DOI:** 10.1186/s12884-023-05517-5

**Published:** 2023-03-24

**Authors:** Tafere Birlie Ayele, Yikeber Abebaw Moyehodie

**Affiliations:** 1 Department of Integrated Emergency Surgery and Obstetrics, College of Medicine and Health Sciences, Bahir Dar University, Bahir Dar, Ethiopia; 2 Department of Statistics, Debre Tabor University, Debre Tabor, Ethiopia

**Keywords:** Gestational age, Logistic regression, Preterm, Prevalence, Mothers

## Abstract

**Backgrounds:**

Preterm birth is defined as babies born alive before 37 weeks of pregnancy or fewer than 259 days since the first day of a woman’s last menstrual period. Globally, 14.84 million babies were preterm births. Preterm infants are at risk for specific diseases related to the immaturity of various organ systems. This study aimed to assess the prevalence of preterm birth and associated factors among mothers who gave birth in public hospitals of east Gojjam zone, Ethiopia.

**Methods:**

An institutional-based cross-sectional study was conducted from April 1 up to June 30, 2021, in public hospitals in the east Gojjam zone. Systematic random sampling was used. Data were collected through structured questionnaires, patient interviews and patient card reviews. We used binary logistic regression analysis with 95% CI and P-value < 0.05 to identify the significant factors with preterm birth.

**Results:**

Out of 615 mothers, 13.2% gave a preterm birth. Antenatal care (AOR = 2.87; 95% CI = (1.67, 5.09)), educational status of mother (AOR = 2.79; 95% CI = (1.27, 6.67)), husband educational status(AOR = 2.11; 95% CI = (1.10, 4.18)), Average monthly family income(AOR = 1.95; 95% CI = (1.05, 3.75)),family size(AOR = 0.15; 95% CI = (0.03, 0.67)), multifetal gestation (AOR = 3.30; 95% CI = (1.29, 8.69), having Premature Rupture Of Membrane (AOR = 6.46; 95% CI= (2.52, 18.24)), history of chronic illness (AOR = 3.94; 95% CI = (1.67, 9.45)), being HIV positive(AOR = 6.99; 95% CI= (1.13, 44.65)), Ante-Partum Hemorrhage (AOR = 3.62; 95% CI= (1.12, 12.59)), pregnancy Induced Hypertension (AOR = 3.61; 95% CI= (1.19, 11.84)), mode of delivery (AOR = 7.16; 95% CI = (2.09, 29.29)), and onset of labor (AOR = 0.10; 95% CI = (0.03, 0.29)) were found to be significantly associated with preterm birth.

**Conclusions:**

antenatal care, educational status of the mother, husband’s educational status, family income, family size, multifetal gestation, Premature Rupture of the membrane, history of chronic illness, being HIV positive, Ante-Partum Hemorrhage, pregnancy Induced Hypertension, mode of delivery, and the onset of labor were found to be significantly associated with preterm birth. To minimize the proportion of preterm birth focusing on this important variables, timely identification of obstetric complications, strengthening early screening of HIV and high-risk pregnancies like multiple gestations, PIH and APH were important.

## Background

Preterm birth (PTB) is defined as babies born alive before 37 weeks of pregnancy or fewer than 259 days since the first day of a woman’s last menstrual period [1]. Globally, 14.84 million babies were preterm births. The majority of these births occurred in Asia and sub-Saharan Africa [2]. Preterm birth is a global problem, with 60% of preterm births occurring in Africa and South Asia. On average, 12% of babies born in the poorest countries are premature, compared with 9% in higher-income countries [3]. Direct complications of preterm birth account for one million deaths yearly, and preterm birth is a risk factor in over 50% of all neonatal deaths [4].

Preterm infants have a higher risk of developing specific diseases due to the immaturity of various organ systems and the causes of preterm birth. As a result of their prematurity, preterm babies are subjected to serious illnesses or deaths during their neonatal period. In the absence of appropriate treatment, survivors are more likely to suffer a lifelong disability and a compromised quality of life. Prematurity complications are the leading cause of neonatal death and the second leading cause of death among children under 5 years old. [5]. Preterm birth complications were the leading cause of death in children younger than 5 years of age globally, accounting for approximately 16% of all deaths and responsible for 35% of deaths among newborn babies [6]. Prematurity now takes the first place for neonatal intensive care unit (NICU) admission, longer hospital stay, the second leading cause of death in children under 5 years, and the single most important direct cause of death in the critical first month of life of infants [7, 8].

In low- and middle-income (LMIC) countries, preterm births account for more than 60% of all births, and the rate has steadily increased. Despite this high preterm birth rate, it is challenging to determine the trend of preterm birth in the majority of low-income economies due to a lack of accurate data [9]. Sub-Saharan African countries have a high preterm birth rate: 23.7% in Nigeria [10], 18.3% in Kenya [11], and 16.3% in Malawi [12]. The overall prevalence of preterm birth in Ethiopia was 10.48% [13]. There is a high rate of infant mortality (48 deaths per 1,000 live births) and neonatal mortality (29 deaths per 1,000 live births) and complication of preterm birth is a major risk factor of this mortality [14]. In the Amhara region, the prevalence of preterm birth was 11.41% [15].

The complication of infants born at preterm gestational age results in a trivial cost to the health sector, parents, and society. The prediction and prevention of preterm birth is a major health care priority [16]. Global efforts to further reduce child mortality demand urgent action to address preterm birth. However, it is a complex multifactorial process associated with diverse pathogenic mechanisms and the prevalence of preterm delivery is one of the strongest predictors of neonatal mortality in our country. During the neonatal period, preterm babies are at a higher risk of serious illness or death. Those who survive preterm delivery without proper care are at great risk of chronic impairment and poor quality of life [17].

Previous studies conducted in different regions indicated that several risk factors were identified for preterm birth. This includes having a previous preterm birth, having a short cervix, short intervals between pregnancies, and certain pregnancy complications (including multiple pregnancy, pregnancy-induced hypertension, premature rupture of the membrane, and vaginal bleeding), chronic illness, educational status, Multiple pregnancies, maternal age, residing in rural areas, antenatal care visits, being HIV positive, family number, and income [15, 18–26].

PTB is a major public health problem. However, in most low-income countries, including Ethiopia, little emphasis is given to PTB intervention as a means of reducing infant mortality. Health care providers or other stakeholders who worked in this public health problem need data related to common factors associated with preterm birth. However, in our country, the studies conducted about this problem were minimal. Although few studies have been conducted in some areas of Ethiopia, the magnitude and possible risk factors of PTB vary by area. A number of methodological issues are addressed in our study (sample size calculation, sampling technique, and multicenter study area) that were not considered in previous studies. Studying a large region is essential to designing effective interventions and programs in public health. Most of the previous study focused mainly on the prevalence of PTB, rather than associated factors of PTB. In addition, different cultures and socioeconomic statuses within a society have varied factors of preterm birth. Therefore studying in different and multicenter settings was important. Determining preterm birth prevalence and associated factors greatly guides health professionals and health policymakers to identify indicators for monitoring preterm birth strategy and applying necessary preventive and appropriate measures to decrease preterm birth. Moreover, the study area’s magnitude of preterm births and associated factors were unknown. Therefore, this study was conducted to assess the prevalence of preterm birth and to identify factors associated with preterm birth in public hospitals in the east Gojjam zone.

## Methods

### Study design

The hospital based cross-sectional study design was conducted using interviewer administered questionnaire from April 1 up to June 30, 2021. Additional information was obtained from medical records of the mothers and babies.

### Study area

The study was conducted in public hospitals in the east Gojjam zone, Amhara National Regional State, Ethiopia. Debre Markos city is a zonal administrative city. It covers 14,010 square kilometers and is divided into 18 administrative districts, further subdivided into 49 urban and 392 rural kebeles, the smallest administrative units [27]. The East Gojam zone is located in northwest Ethiopia, which is 265 Km far from Bahirdar, the capital city of Amhara region, 299 km from Addis Ababa, the country’s capital. The Oromia region borders the zone on the south, West Gojjam on the west, South Gondar on the north, and South Wollo on the east. With a population of 3.8 million, the East Gojjam Zone has 21 Woreda, 480 Kebeles, 10 government hospitals (1 referral hospital, 9 primary hospitals), 102 health centers, and 423 health posts [28–30]. This study was conducted in five randomly selected public hospitals. The randomly selected public hospitals were Shegaw Motta general hospital, Bichena primary hospital, Dejen primary hospital, Lumame primary hospital, and Debre Markos referral hospital. All these health institutions are currently providing maternal and child health care services.

### Participants

All mothers who gave birth in randomly selected public hospitals in the east Gojjam zone during the study period were our study population. Mothers who gave birth and had known either LNMP or had early ultrasound (before 24 weeks) diagnosis were included for this study. Mothers who were deaf and comatose, had unknown last normal menstrual period (LNMP) and had no early ultrasound (before 24 weeks) were excluded from this study.

### Sampling technique and sample size determination

A systematic sampling technique was used. The sample was arranged based on the three-month patient flow before the data collection period by referring to the hospital’s delivery registration book/ record. To calculate K, the summation of the three months delivery report for the hospital was 1698.Then K = N/n, 1698/615 = 2.8 ≈ 3. Where k = interval, N = total population, n = sample size. Every third mother’s was interviewed, gestational age of the newborn was calculated based on the mothers LNMP or first-trimester ultrasound result, in estimating gestational week, when there are extra days it was counted to the near lowest gestational age. The next participant was taken when the selected study participant was not eligible for the study.

The sample size was determined by using a single population proportion formula by considering the prevalence (p) of preterm birth = 15.5%, which was obtained from the previous study in Ethiopia [17], 95% confidence interval ($z\frac{\alpha }{2}$=1.96), and level of precision (d) = 0.03.

1 $$n=\frac{\left({{Z}_{\frac{\alpha }{2}}}^{2}\right)\left(p\right)(1-p)}{{d}^{2}}$$

2 $$n=\frac{{ 1.96}^{2}\left(0.155\right)\left(0.845\right)}{\left({0.03}^{2}\right)}=559$$

Finally, after taking a 10% non-response rate the total sample size (n) was 615. An average delivery report for a month before the actual data collecting period was computed for each hospital by analyzing the client’s registration book to distribute the sample size proportionally to each hospital. The sample size was then proportionally allocated to each hospital. Finally, the immediate postnatal mother with her baby had been selected every three intervals using a systematic sampling technique.

### Data collection

Data were collected and extracted by reviewing medical records of mother’s .The data collectors were four fourth year undergraduate midwives students from Debre Markos University. One staff midwife from each hospital was assigned to supervise the data collection process. The data collection process was supervised by both the principal investigator and the supervisor. Information collected from the mother included Residency, Age, Religion, Education status, Occupation, Marital status, Average monthly Average monthly family income, Antenatal Care (ANC), inter pregnancy interval parity, Educational Status of husband, Ante-Partum Hemorrhage (APH), Premature Rupture Of Membrane (PROM), Pregnancy Induced Hypertension (PIH), multiple pregnancies, polyhydramnios, anemia, cardiac disease, hypertension, HIV status, Modern contraceptive use before current pregnancy, Urinary Tract Infection (UTI) and malaria were the predictor variable for this study. Anemia was defined as an HGB level below 11gm/dl (HCT < 33%). Obstetric complications was defined as challenges or problems that happen during labor or delivery. PIH was defined clinically as a blood pressure of > 140/90 mmHg after 20 weeks of gestation with or without proteinuria and/or edema as diagnosed and documented by the attending clinician. APH was defined as any vaginal bleeding in the mother after 24 weeks of gestation as documented in the records by the attending clinician. UTI was defined as a documented clinical/laboratory diagnosis of UTI any time during the pregnancy and/or a positive history of treatment of burning sensation with micturition as reported by the mother. Birth to pregnancy interval was defined as the time between the start of the index pregnancy and the preceding live birth. Last normal menstrual period was defined as the date of the starting of last normal menstruation the women had to index pregnancy. Preterm birth was defined as a newborn with a gestational age of 28 weeks to less than 37 weeks. To assure the quality of data, the questionnaire was pre-testing on 31 mothers in public hospitals in the east Gojjam zone, and the questionnaire’s fitness was confirmed from pre-testing. One-day practical training on how to collect data was given to the data collectors and the supervisor before data collection. During the data collection period, the collected data were reviewed, checked for completeness, and signed by the supervisor at the end of each day.

### Data processing and analysis

All questionnaires were checked, coded, and entered into the SPSS version 25 software packages, which were then analyzed using R 4.1.3. The data were presented using frequency tables and graphs. The relevant determinants of preterm birth were identified using binary logistic regression. The researchers used both bivariable and multivariable analyses. In the bivariable analysis, independent variables with a p-value less than 0.25 were chosen for the multivariable analysis. An adjusted odds ratio with a 95% confidence level was used to examine the degree of relationship between independent and dependent variables, and variables with a p-value of 0.05 were considered statistically significant.

## Results

### Socio-demographic characteristics the of the respondents

All participants completed the interview (100% response rate). The majority of the respondents 358 (58.2%) were rural residents. The majority of the study participants 360 (58.5%) were between 25 and 34 years old. The entire respondent belongs to Amhara by ethnicity, and 516 (96.1%) were Orthodox Christians in religion. Regarding the marital status of the respondents, the majority of them 596(96.9%) were married. 253 (41.1%) were have no formal education, 217(35.3%) of the respondents were taken education in primary school, and 253(41.1%) of them can only read and write, and the remaining 145 (23.6%) were secondary and above education level. The occupational status of most of the respondents 228(37.1%) were farmers, 144 (23.4%) of the respondents were housewives, 150 (24.4%) of the respondents were employers, and the remaining respondents had other occupations (labor work, no work, students, work-seekers). 264 (42.9%) of the husband were have no formal education. 264(42.9%) of the respondent’s family average monthly income is less than or equal to 3000 Birr and 212(34.5%) of the respondent’s family average monthly income is average greater than 5000 Birr. The majority of the respondents, 415 (67.5%) had between 3 and 5 family members (Table 1).

**Table 1:** Socio-Demographic Characteristics of the study participants in public hospitals of east Gojjam zone, Ethiopia, 2021 from April to June

Variables	Categories	Frequencies	Percent (%) (N = 615)
Residence	Urban	257	41.8
	Rural	358	58.2
Age	15–24	182	29.6
	25–34	360	58.5
	> 35	73	11.9
Marital status	Married	596	96.9
	Others(Divorced, Widowed, and Single)	19	3.1
Religion	Orthodox	591	96.1
	Others(protestant, and Muslim)	24	3.9
Educational status of Mothers	no formal education	253	41.1
	primary education	217	35.3
	secondary and above	145	23.6
Occupational status of the respondents	Housewife	144	23.4
	Employer	150	24.4
	Farmer	228	37.1
	Other	93	15.1
Educational Status of the husband	no formal education	264	42.9
	primary education	139	22.6
	secondary and above	212	34.5
Average monthly family income	<=3000 Birr	264	42.9
	3001–5000 Birr	139	22.6
	> 5000 Birr	212	34.5
Family size	< 3	174	28.3
	3–5	415	67.5
	> 5	26	4.2

### Obstetric and medical-related characteristics

Most of the respondents (96.3%) had ANC follow up and 56.1% of the respondent had at least 4 visits. The majority of the respondents used modern contraceptives (94.8%) before their pregnancy, and the majority of mothers had their pregnancy wanted and planned (99.3%). More than two-thirds of the respondents had birth-to-pregnancy intervals greater than or equal to 36 months (83.5%). Labor spontaneously started in 87.8% of the respondents. Three-quarters of the respondents (74.3%) gave birth by SVD. Out of the total respondents, 595(96.7%) were tested for HIV status, and 36 (5.7%) respondents were HIV positive. (133) 21.6% of women had APH, (142) 23.1%, of women had PIH, (70) 11.4%, of mothers had multiple pregnancies, and women had Polyhydramnios (10) 1.6%. 105(17.1%) of the respondents had a history of chronic illness. 70(11.4%) of mothers had urinary tract infection (Table 2).

**Table 2:** Obstetric and medical related characteristics of the study participants in public hospitals of east Gojjam zone, Ethiopia, 2021 from April to June

Variables	Categories	Frequency	Percent (%) (N = 615)
ANC visit	Yes	592	96.3
	No	23	3.7
ANC follow up	< 4 follow up	345	56.1
	>=4 follow up	270	43.9
Pregnancy status	Planned	611	99.3
	Unplanned	4	0.7
Modern contraceptive use before current pregnancy	Yes	583	94.8
	No	32	5.2
Birth to pregnancy interval in months	< 36	65	16.5
	≥ 36	329	83.5
Mode of delivery	SVD	457	74.3
	CS or Instrumental delivery	158	25.7
Onset of labor	Spontaneous	540	87.8
	Induced	75	12.2
History of HIV testing	Yes	595	96.7
	No	20	3.3
Status of HIV (n = 595)	Positive	36	5.7
	Negative	559	91.1
	Un known	20	3.3
Blood RH factor	Positive	555	90.2
	Negative	60	9.8
PROM	Yes	219	35.6
	No	396	64.4
HGB (mg/dl)	< 11	29	4.7
	≥ 11	586	95.3
APH	Yes	133	21.6
	No	482	78.4
PIH	Yes	142	23.1
	No	473	76.9
Multiple pregnancies	Yes	70	11.4
	No	545	88.6
Polyhydramnios	Yes	10	1.6
	No	605	98.4
History of Chronic illness	Yes	105	17.1
	No	510	82.9
Urinary Tract Infection.	Yes	70	11.4
	No	545	88.6

### The proportion of preterm birth

**Fig. 1:**
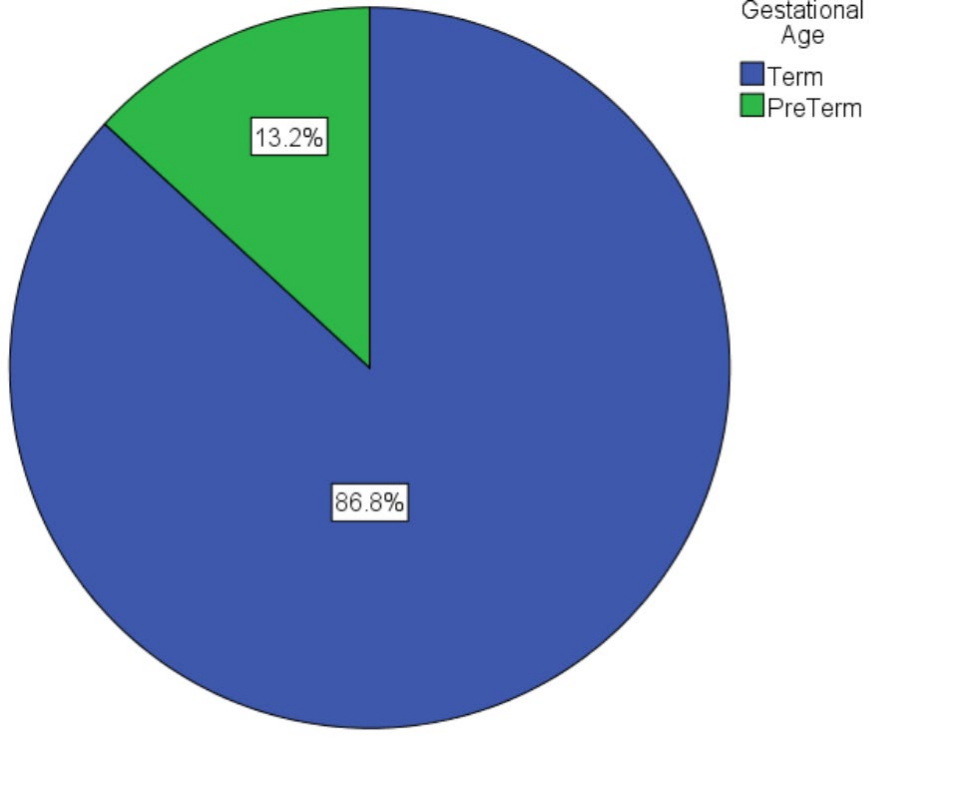
proportion of preterm birth in the east Gojjam zone

The proportion of preterm birth in this study was found to be 13.2% (CI: 0.11, 0.16) (Fig. 1).

### Factors associated with preterm birth

All independent variables were analyzed using binary logistic regression with the dependent variable preterm birth and those, which were significant at a p-value of < 0.25 were transferred to multivariable logistic regression analysis. The variable with a p-value < 0.05 was significant.

In bivariable analysis ANC follow-up, educational status of mother, husband educational status, family income, marital status, occupation, family size, PROM, being HIV positive, obstetric complication, APH, PIH, history of chronic illness, multifetal gestation, RH factor, pregnancy status, mode of delivery, onset of labor, and UTI were found to be significantly associated with pre term birth. In multivariable binary logistic regression analysis ANC follow up (AOR = 2.87; 95% CI = (1.67, 5.09)), educational status of mother (AOR = 2.79; 95% CI = (1.27, 6.67)), husband educational status(AOR = 2.11; 95% CI = (1.10, 4.18)), Average monthly family income(AOR = 1.95; 95% CI = (1.05, 3.75)),family size(AOR = 0.15; 95% CI = (0.03, 0.67)), multifetal gestation (AOR = 3.30; 95% CI = (1.29, 8.69), having PROM(AOR = 6.46; 95% CI= (2.52, 18.24)), history of chronic illness (AOR = 3.94; 95% CI = (1.67, 9.45)), being HIV positive(AOR = 6.99; 95% CI= (1.13, 44.65)), APH(AOR = 3.62; 95% CI= (1.12, 12.59)), PIH (AOR = 3.61; 95% CI= (1.19, 11.84)), mode of delivery (AOR = 7.16; 95% CI = (2.09, 29.29)), and onset of labor (AOR = 0.10; 95% CI = (0.03, 0.29)) were found to be statistically significant at p-value of < 0.05 (Table 3).

**Table 3:** Factors associated with preterm birth among study participants in public hospitals of east Gojjam zone, Ethiopia, 2021 from April to June

Variables		N = 615 Preterm		COR (95% CI)	AOR(95% CI)
		Yes	No		
**ANC** **follow up**	< 4	60	285	2.50( 1.50, 4.31)	2.87(1.67, 5.09)
	≥ 4	21	249	1	1
**Marital status**	Married	74	522	0.24(0.09, 0.67)	0.44(0.06, 3.92)
	Others(Divorced, Widowed, and Single)	7	12	1	1
**Education status of mothers**	no formal education	52	201	3.91( 1.95, 8.73)	2.79 (1.27, 6.67)
	primary education	20	197	1.53( 0.70, 3.64)	1.53( 0.65, 3.86)
	secondary and above	9	136	1	1
**Husbands education status**	no formal education	45	153	3.35( 1.88, 6.24)	2.11( 1.10, 4.18)
	primary education	19	186	1.18( 0.59, 2.36)	0.88(0.42, 1.85)
	secondary and above	17	195	1	1
**Average monthly family income**	<=3000 Birr	52	212	2.81( 1.60, 5.16)	1.95(1.05, 3.75)
	3001–5000 Birr	12	127	1.08( 0.49, 2.33)	0.86( 0.38, 1.94)
	> 5000 Birr	17	195	1	1
**Family size**	< 3	30	144	0.47(0.19,1.23)	0.51(0.09, 2.68)
	3–5	43	372	0.26(0.11, 0.66)	0.15(0.03, 0.67)
	> 5	8	18	1	1
Occupational status of the respondents	Housewife	26	118	1.64(0.79, 3.64)	1.42( 0.63, 3.35)
	employer	8	142	0.42(0.157, 1.08)	0.61( 0.22, 1.64)
	Farmer	36	192	1.39(0.69, 3.00)	0.60( 0.27, 1.41)
	Other	11	82	1	1
**Mode of delivery**	SVD	69	388	2.16(1.18, 4.30)	7.16(2.09, 29.29)
	CS or Instrumental delivery	12	146	1	1
**Multifetal gestation**	Yes	23	47	4.12(2.30, 7.20)	3.30(1.29, 8.69)
	No	58	487	1	1
PROM	Yes	48	171	3.09(1.92, 5.02)	6.46(2.52,18.24)
	No	33	363	1	1
APH	Yes	44	89	5.94(3.64,9.78)	3.62(1.12, 12.59)
	No	37	445	1	1
PIH	Yes	52	90	8.85(5.36, 14.85)	3.61(1.19, 11.84)
	No	29	444	1	1
**History of Chronic illness**	Yes	35	70	5.04(3.03, 8.37)	3.94( 1.67, 9.45)
	No	46	464	1	1
**UTI**	Yes	26	44	5.26(2.99, 9.18)	2.41(0.37, 16.42)
	No	55	490	1	1
**Status of HIV(n = 595)**	Positive	15	21	5.83(2.8, 11.86)	6.99(1.13, 44.65)
	Negative	61	498	1	1
Pregnancy status	Planned	79	532	0.15(0.02, 1.25)	0.19(0.01, 35.87)
	Unplanned	2	2	1	1
RH Factor	positive	79	476	4.81(1.46, 29.73)	1.82(0.31,16.75)
	negative	2	58	1	1
**onset of labor**	Spontaneous	50	490	0.14(0.08, 0.25)	0.10(0.03, 0.29)
	Induced	31	44	1	1

*Abbreviations:* 1 = Reference, *ANC* Antenatal Care, *AOR* Adjusted Odds Ratio, *APH* Ante-Partum Hemorrhage, *CI* Confidence Interval, *COR* Crud Odds Ratio, *CS* Cesarean Section, *HIV* Human Immune Deficiency Virus, *PIH* pregnancy Induced Hypertension, *PROM* Premature Rupture of Membrane, *SVD* Spontaneous Vaginal Delivery, *UTI* Urinary Tract Infection

## Discussion

This study was conducted to assess the magnitude of preterm birth and its associated factors in public hospitals in the east Gojjam zone. During the study period, the overall proportion of preterm birth was found to be 13.2%. The finding was greater than the preterm birth rate for the world (9.8%) and North America (9%) [2, 31]. It was also more than the prevalence of preterm birth in Ethiopia, 10.1%, which was reported by the Global Action Report on Preterm Birth [32]. Compared with cross-sectional studies conducted in our country the finding was found to be in line with the finding conducted in Debre Tabor town health institutions, which was 12.8%, and with the finding conducted in Axum and Adwa town public hospitals in which the prevalence of preterm birth was 13.3% [33, 34].

The proportion of preterm birth in this study however was higher than another study conducted in our country at Gondar town health institutions, which reported a prevalence rate of 4.4% [18]. This discrepancy may be due to differences in exclusion criteria for multiple pregnancies. In our study, mothers with multiple pregnancies were included, whereas these mothers were excluded from the mentioned study. Therefore, a lower rate was expected in their study, as over distention of the uterus as in multiple pregnancies and polyhydramnios is one of the scientifically explained causative factors for preterm labor. The prevalence of preterm was also higher than the prevalence of most developed nations. The preterm birth rate from the study conducted in Sweden which was estimated to be 5.03% is good evidence [35]. The low preterm birth rate in developed nations like Sweden may be due to high socio-demographic status of the population and improved preconception and ANC services, which are important in early identifying and preventing risk factors. The proportion of preterm birth in this study was found to be lower than in some studies conducted in low and middle-income countries.

The study conducted in Malawi shows that the prevalence of preterm birth was 16.3% [12]. This higher prevalence of preterm birth in Malawi may be due to the country’s higher HIV infection rate, where one in four women are HIV positive. In Brazil, the prevalence of preterm birth among young women attending public hospitals was 21.7% which was higher compared to this study [36]. The discrepancy may be due to variation in the study population. Only parturient mothers aged 15–24 were included in their study. A similar study conducted in Nigeria reported the prevalence of preterm birth of 16.9% [37], which was also higher than this study. This variation is maybe because of the difference in the study area where their study was at a referral hospital with referrals of more complicated cases from other general hospitals.

The current study’s finding was lower than those conducted in Kenya National Hospital and Jemma University Specialized Hospital, which reported the prevalence of preterm birth was 20.2% and 25.9%, respectively [23, 38]. This variation might be due to the difference in the study time, reflecting that FMOH has currently improved maternal health care service. Another possible reason for this variation might be due to differences in the study area, the study done in Kenya and Jimma indicates that the high prevalence of alcohol consumption and substance intake during pregnancy may be the contributing factors to the increased magnitudes of preterm birth.

Mothers with less than 3000 birr Average monthly family income were 1.95 times more likely to develop preterm than those with greater than 5000 birr family income. This study was in line with the study in SSA [26, 39],which shows Average monthly family income positively affected preterm. This might be due to financial insecurity, psychosocial stress, and low health care utilization. This study was not in line with the studies in Ethiopia, which show Average monthly family income was not associated with preterm mothers [18].

Mothers from three up to five family members were 0.15 times less likely to develop/give preterm as compared to mothers from greater than five family members. This study was in line with the study in Ethiopia [17], which shows family members had a significant positive effect on preterm birth.

The education status of mothers were significant factors of preterm. Mothers who had no formal education were 2.79 times more likely to develop/give preterm than mothers who had secondary and above education levels. This study was in line with the studies in Sub-Saharan African and European countries [39–41], which show education had been associated with preterm birth. This study was not in line with the studies in Ethiopia and Kenya [38, 42], which show education statutes had no significant effect on preterm.

Mothers who had husbands who had no formal education were 2.11 times more likely to develop/give preterm than mothers who had husbands with secondary and above education levels. This study is in line with the previous study in Iran [43], which shows a significant association between husbands’ education level and preterm birth.

Mothers with APH were 3.62 times more likely to have a preterm birth than mothers without APH. This finding is in line with the study conducted in Kenya, Ethiopia, and East Africa [38, 44, 45]. This suggests that obstetric problems caused by APH may significantly impact the occurrence of PTB. This could be due to decreased placental blood flow, impacting the mother-fetus exchange of nutrients and oxygen. As a result, fetal growth would be slowed, and the chance of PTB would be increased.

Mothers with multifetal gestation were 3.30 times more likely to have a preterm birth than mothers without multifetal gestation. This finding is in line with the study conducted in Ethiopia, Korea, Greek [46–48]. It might be since multiple pregnancies are more likely to be associated with a variety of problems, including preeclampsia, PROM, and polyhydramnios, all of which could contribute to iatrogenic PTB. Furthermore, this could be related to uterine overstretching and deciding to terminate the pregnancy before it reaches term.

Mothers who delivered with SVD were 7.16 times more likely to develop preterm as compared to mothers delivered with CS or Instrumental delivery. This finding is in line with the study conducted in Ethiopia [49], which shows delivered with SVD had a positive significant effect on preterm. This study is not in line with [50], which shows women delivering by previous cesarean section had a significantly higher risk of preterm birth when compared to women with vaginal delivery.

Mothers with spontaneous labor were 0.10 times less likely to have preterm birth as compared to mothers with induced labor. This finding is in line with the study conducted in Ethiopia [49], which shows spontaneous labor had a negative impact on preterm mothers. In addition, this study is in line with the study conducted in Ethiopia [51], which shows Labor status was associated with preterm birth.

History of chronic illness was significantly associated with the outcome variable, mothers who had a history of chronic illness were 3.94 times more likely to give preterm birth than mothers who had no chronic illness history. This finding is in line with a study conducted in Ethiopia [18, 44, 49], which shows chronic illness positively associated with preterm. This might be due to maternal illnesses that impede or impair the placental transfer of oxygen and nutrients to the developing fetus in the uterus can raise the chance of preterm birth.

This study revealed a significant association between pregnancy-induced hypertension and preterm birth. Mothers who had complications PIH were 3.61 times increased risk of having a preterm birth than those mothers without this problem during the index pregnancy. This finding is in line with the study conducted in East Africa, Ethiopia, Nigeria, Iran, Ghana, and Kenya [18, 38, 45, 46, 51–55], which shows PIH had significant effect on preterm birth. This might be due to the vascular damage of the placenta caused by PIH, which results in preterm labor and delivery.

Another significant association was found between mothers who had premature rupture of membranes (PROM) and preterm birth. Mothers with PROM were 6.46 times more likely to have a preterm birth than their counterparts. This finding was in line with the previous findings in Tehran, Iran, Ethiopia, East Africa, Nigeria [7, 26, 46, 53, 56], showing a significant association between PROM and preterm. This might be because in the absence of any clinical intervention labor will spontaneously initiate within hours after term PROM and within a week after preterm PROM in the majority of the cases.

In this study, the ANC follow up were significantly associated with the outcome variable preterm birth. Mothers with < 4 times ANC follow-up in the index pregnancy were 2.87 times more likely to have a preterm birth than mothers who had the ANC visits ≥ 4 times. This finding is in line with a study conducted in Ghana, Nigeria teaching hospitals, and western Ethiopia [20, 44, 52, 54, 57], which shows a significant association between ANC visits and preterm. WHO recommends at least four ANC visits, this will assure the quality of care and early detection of high-risk pregnancies which result in the prevention and proper management of obstetric complications. This may happen regularly. The benefits of an ANC visit include health promotion, early detection, and treatment of obstetric problems. However, this study is not in line with the study conducted in Ethiopia [18], which shows ANC follow-up had no significant effect on preterm.

Being HIV positive was significantly associated with preterm birth. HIV-positive mothers were 6.99 times more likely to give preterm compared to HIV-negative mothers. This finding is in line with a study in Malawi and Ethiopia [12, 18, 41, 58, 59], which shows a significant association between prevalence of HIV and preterm birth. This might be due to the drug effect and immunity of the mother as risk factors for preterm birth. This study was not in line with studies conducted in Botswana, and Malawi [60, 61]. This might be due to the drug’s impact and the mother’s immunity to premature birth.

In contradiction to the previous studies in our study, age, marital status, RH factor, Pregnancy status, and UTI [42, 62, 63]. This difference can be due to differences in the study area, design, period, population, and culture differences.

### Limitation of the study

Despite efforts to reduce recollection biases by informing local events, there may be a recall bias. When ultrasound facilities are not accessible, the menstrual history and clinical examination are used to confirm gestational age and which may subject to considerable error. Another drawback of our study is using secondary data for some variables.

## Conclusion

The proportion of preterm birth in public hospitals in the east Gojjam zone is 13.7%. number of ANC, educational status of mother, husband educational status, family income, family size, occupation, multifetal gestation, having PROM, history of chronic illness, being HIV positive, APH, PIH, mode of delivery, and onset of labor were found to be significantly associated with preterm birth. As a result, focusing on these important variables would reduce the number of premature births. Furthermore, it was suggested that educating the community about the importance of ANC service utilization of mothers and preventing preterm birth be strengthened. Moreover, strengthening early screening of HIV and high-risk pregnancies like multiple gestations, PIH and APH were important to prevent preterm.

## Data Availability

The datasets used and/or analyzed during the current study are available from the corresponding author upon reasonable request.

## Abbreviations

ANC: Antenatal Care
AOR: Adjusted Odds Ratio
APH: Ante-Partum Hemorrhage
CHTN: Chronic hypertension
CI: Confidence Interval
COR: Crud Odds Ratio
CS: Cesarean Section
DCSH: Debre Markos comprehensive specialized hospital
DM: Diabetes mellitus
EDHS: Ethiopian Demographic and Health Survey
HCT: Hematocrit
HGB: Hemoglobin
HIV: Human Immune Deficiency Virus
IESO: Integrated Emergency Surgery and Obstetrics
LNMP: last normal menstrual period
LMICS: Lower and Middle-Income Countries
MDG: Millennium Development Goal
NICU: Neonatal Intensive Care Unit
PIH: pregnancy Induced Hypertension
PROM: Premature Rupture Of Membrane
PTB: Preterm Birth
SD: Standard Deviation
SPSS: Statistical Package of Service solution
SVD: Spontaneous Vaginal Delivery
UTI: Urinary Tract Infection
WHO: World Health Organization
